# ACSM6 overexpression indicates a non-inflammatory tumor microenvironment and predicts treatment response in bladder cancer: results from multiple real-world cohorts

**DOI:** 10.3389/fphar.2023.1222512

**Published:** 2023-06-22

**Authors:** Zhiwei Li, Yiyan Yao, Tiezheng Qi, Zuowei Wu, Dingshan Deng, Bolong Liu

**Affiliations:** ^1^ The Second Affiliated Hospital, Department of Urology, Hengyang Medical School, Unversity of South China, Hengyang, Hunan, China; ^2^ Xiangya School of Medicine, Central South University, Changsha, Hunan, China; ^3^ Department of Interventional Radiology, Third Xiangya Hospital, Central South University, Changsha, China; ^4^ Department of Urology, National Clinical Research Center for Geriatric Disorders, Xiangya Hospital, Central South University, Changsha, Hunan, China; ^5^ National Clinical Research Center for Geriatric Disorders, Xiangya Hospital, Central South University, Changsha, Hunan, China; ^6^ The First Affiliated Hospital, Department of Andrology, Hengyang Medical School, University of South China, Hengyang, China

**Keywords:** ACSM6, bladder cancer, tumor microenvironment, immunotherapy, chemothearpy bladder cancer, chemotherapy

## Abstract

**Background:** ACSMs play critical roles in lipid metabolism; however, their immunological function within the tumor microenvironment (TME) remains unclear, especially that of ACSM6. In this study, we investigate the latent effect of ACSM6 on bladder cancer (BLCA).

**Methods:** Several real-world cohorts, including the Xiangya (in-house), The Cancer Genome Atlas (TCGA-BLCA), and IMvigor210 cohorts, with TCGA-BLCA cohort serving as the discovery cohort were compared. We investigated the potential immunological effects of ACSM6 in regulating the BLCA tumor microenvironment by analyzing its correlation with immunomodulators, anti-cancer immune cycles, immune checkpoints, tumor-infiltrating immune cells, and the T-cell inflamed score (TIS). Additionally, we assessed the precision of ACSM6 in predicting BLCA molecular subtypes and responses to several treatments using ROC analysis. To ensure the robustness of our findings, all results were confirmed in two independent external cohorts: the IMvigor210 and Xiangya cohorts.

**Results:** ACSM6 expression was markedly upregulated in BLCA. Our analysis suggests that ACSM6 might have significant impact to promote the formation of a non-inflamed tumor microenvironment because of its negative correlation with immunomodulators, anticancer immune cycles, immune checkpoints, tumor-infiltrating immune cells, and the T-cell inflamed score (TIS). Additionally, high ACSM6 expression levels in BLCA may predict the luminal subtype, which is typically associated with resistance to chemotherapy, neoadjuvant chemotherapy, and radiotherapy. These findings were consistent across both the IMvigor210 and Xiangya cohorts.

**Conclusion:** ACSM6 has the potential to serve as a valuable predictor of the tumor microenvironment phenotypes and treatment outcomes in BLCA, thereby contributing to more precise treatment.

## Introduction

According to GLOBOCAN’s 2020 estimates, bladder cancer (BLCA) accounts for approximately 573,278 new cases and 212,536 fatalities worldwide, making it the 10th most frequently diagnosed cancer (Sung et al., 2021). Urothelium carcinoma is the main histological type of BLCA and non-muscle invasive BC (NMIBC) patients account for about 75% of BLCA cases, with obvious heterogeneity and the risk of recurrence and progression to muscle invasive BC (MIBC) (van Rhijn et al., 2009). Therefore, despite the high 5-year survival rate of NMIBC (>90%), most patients have to accept long-term cystoscope monitoring and multiple treatment interventions, resulting in lower quality of life (Catto et al., 2021) and huge medical burden, so BLCA is considered to be the most expensive malignant tumor (Svatek et al., 2014).

Although chemotherapy is commonly used as the first course of treatment for advanced or metastatic BLCA, the discouraging objective response rate and consequent poor five-year survival rate indicate the need for alternative therapies (Liu et al., 2022). Currently, the FDA has only approved FGFR3 inhibitors, PD-1/PD-L1-based immune checkpoint inhibitors, and antibody-drug conjugates for the immunological treatment of BLCA, which are especially suitable for platinum-resistant or non-platinum locally advanced or metastatic urothelial cancer. Recent studies suggest that precision therapy may offer superior efficacy compared with either treatment alone (Morales-Barrera et al., 2020; Chang et al., 2021; Grivas et al., 2021). Considering the limitations of chemotherapeutic drugs and surgery (Feng et al., 2020; Feng et al., 2022a; Feng et al., 2022b), precise and personalized treatment based on biomarkers is also becoming increasingly important for urinary system tumors (Hu et al., 2021a; Hu et al., 2021b; Hu et al., 2022; Cai et al., 2023).

Liposomal coenzyme A synthetase is an enzyme that catalyzes the activation of fatty acids and participates in the 1st step of fatty acid metabolism, which is divided into four categories containing medium-chain acyl-CoA synthetase (ACSM). Primarily, ACSMs are located on human chromosome 16p12, which contains six members: ACSM1–ACSM6. However, the connection between the ACSM family and cancer has rarely been reported, especially for ACSM6.

Through multi-omics analysis, we identified ACSM6 as a novel target for BLCA immunotherapy. We conducted a comprehensive investigation to examine the association between ACSM6 and the tumor microenvironment (TME) in BLCA. Our findings revealed that ACSM6 shaped the non-inflammatory TME in BLCA and enabled the prediction of BLCA molecular subtypes.

## Methods

### Data acquisition and preprocessing of three real-world cohorts

We retrieved BLCA mRNA data using the mRNA expression data (FPKM) values and related clinicopathological messages from TCGA (https://portal.gdc.cancer.gov/). The cohort consisted of 410 BLCA samples and 19 normal urothelial tissue samples. Before analysis, the FPKM values in the TCGA cohort were converted to transcripts per kilobase million (TPM).

The cohort used for validation included patients who underwent surgical treatment for BLCA at Xiangya Hospital. The Xiangya cohort comprised 57 BLCA samples and 13 normal bladder epithelial tissue samples. Data from this cohort were uploaded to the GEO database (GSE188715) (Hu et al., 2021a; Hu et al., 2021b).

The IMvigor210 cohort comprised of patients with BLCA who underwent anti-PD-1 therapy as part of an immunotherapy study. The mRNA expression data and corresponding clinicopathological information were obtained under the Creative Commons 3.0 License (Mariathasan et al., 2018).

### Describing the immunological features of BLCA TME

Anticancer immunity has a lot to do with the cancer immune cycle, the expression of immunomodulatory factors, the level of infiltration of tumor-infiltrating lymphocytes (TILs), and the expression of inhibitory immune checkpoints in the TME. We collected 122 immunomodulatory factors from previous studies and compared differentially expressed immunomodulatory factors, including chemokines, immunostimulatory factors, receptors, and MHC, in the low and high ACSM6 groups (Charoentong et al., 2017; Hu et al., 2021a; Hu et al., 2021b; Liu et al., 2021; Hu et al., 2022; Cai et al., 2023). Subsequently, we investigated the impact of ACSM6 on the cancer immune cycle, which comprises seven crucial steps that determine how the tumor cells take effect in BLCA (Chen and Mellman, 2013). Consequently, we employed five distinct algorithms: TIMER, CIBERSORT-ABS, xCell, quanTlseq, and MCP-counter, to calculate the correlation between the level of TIL infiltration and the expression of ACSM6 in the TME (Newman et al., 2015; Becht et al., 2016; Li et al., 2016; Xu et al., 2018; Finotello et al., 2019; Li et al., 2020). Additionally, we studied the relation between ACSM6 and the corresponding effector genes of the TILs. Furthermore, we examined the correlation between ACSM6 expression and 22 common immune checkpoint inhibitors (ICIs), such as PD-1, PD-L1, CTLA-4, and LAG-3 (Auslander et al., 2018). Finally, we examined the TIS in the TME, its related effector genes, and its effect on the clinical response to immune checkpoint blockade (ICB) (Ayers et al., 2017).

### Predicting BLCA molecular subtypes and treatment response

Owing to the high heterogeneity of BLCA, the treatment response and prognosis of different types are different. There are several molecular classification systems for BLCA, including TCGA, Baylor, UNC, Lund, CIT, MDA and Consensus classification systems (Sassoli et al., 2019). In this study, we used the BLCA subtyping R software package and Consensus MIBC to predict the molecular subtype system and describe the correlation between ACSM6 expression and specific markers of the molecular subtypes. The accuracy of ACSM6 in predicting BLCA molecular subtypes was evaluated using ROC curves. We also evaluated differences in neoadjuvant chemotherapy-related mutations between the high and low ACSM6 groups. Additionally, we explored the prognosis of several treatments such as immunotherapy, targeted therapy, and radiotherapy. Finally, we collected and analyzed drug target genes from the Drug Bank database.

### Statistical analysis

We calculated Pearson’s or Spearman’s coefficients to identify correlations between variables. The *t*-test was used to compare the differences between binary groups for normally distributed variables, whereas the chi-square test or Fisher’s exact test was used for categorical variables. Statistical significance was determined using a two-sided *p*-value of <0.05. We evaluated the accuracy of the molecular subtype prediction using ROC curves. Statistical analyses and visualizations were conducted using R software version 4.2.2.

## Result

### The immunological function of ACSM6 in pan-cancer analysis

**FIGURE 1:**
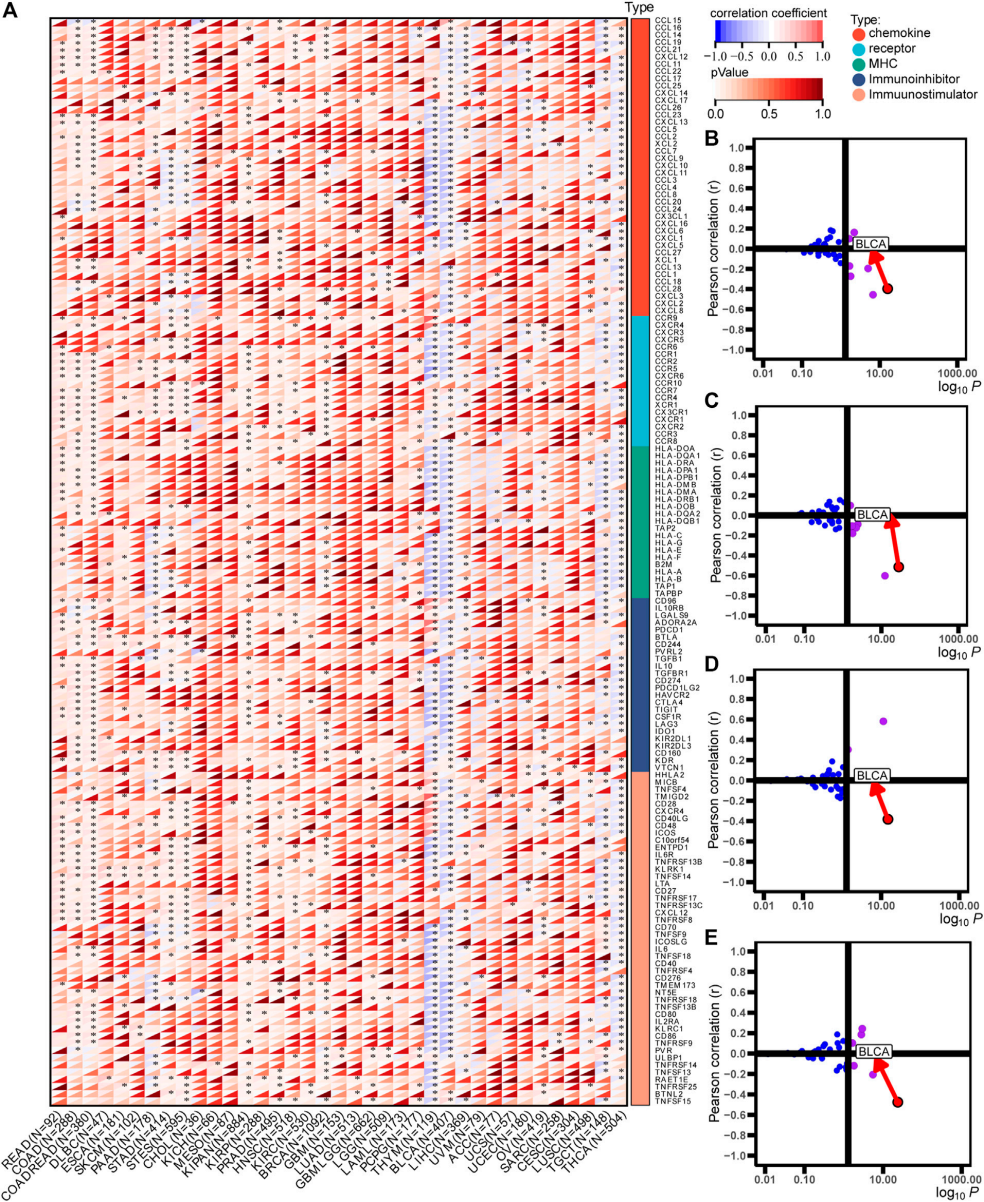
Immunological characteristics in pan-cancers correlated with ACSM6. **(A)** Correlation of ACSM6 with immunomodulators, including chemokines, receptors, MHC, and immunostimulators. **(B–E)** Correlation of ACSM6 expression with PD-L1, CTLA-4, PD-1, and LAG-3.

**FIGURE 2:**
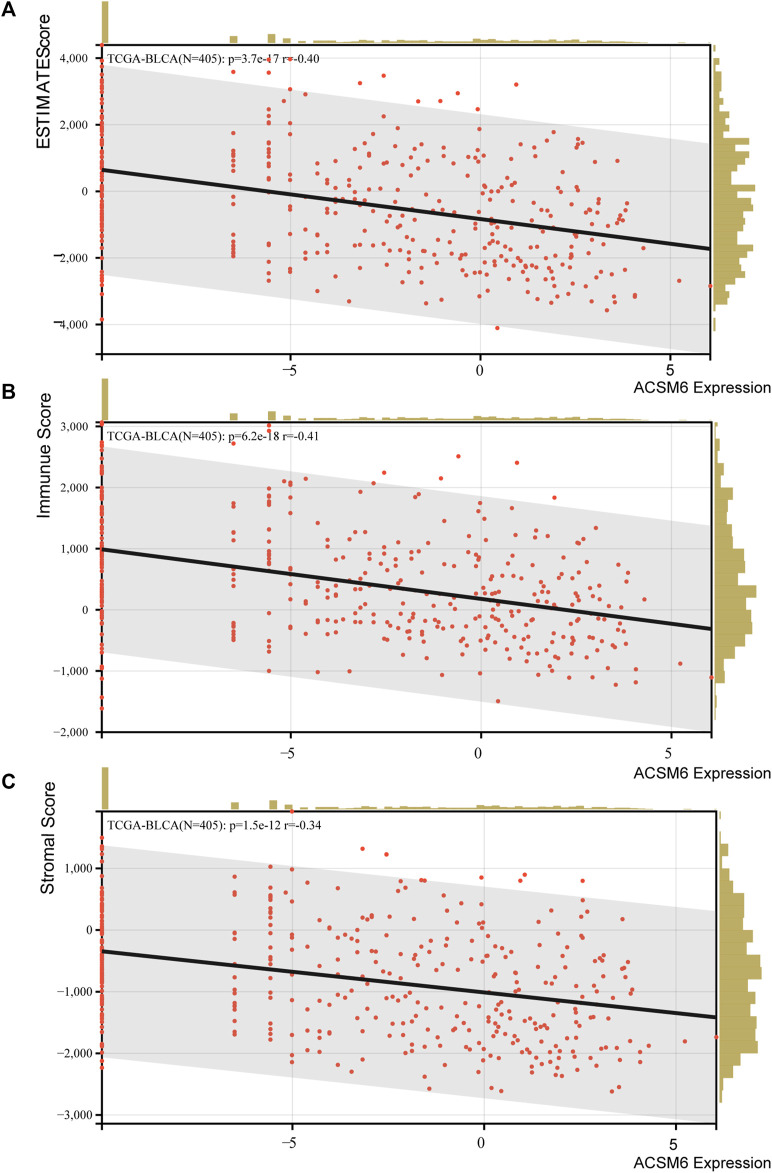
**(A–C)** Correlation between ACSM6 expression and TME scores, including ESTIMATE, immune, and stromal scores.

The immunological role of ACSM6 was determined and the cancer types most affected by ACSM6 were screened using pan-cancer analysis. Figure 1A shows the relationship between ACSM6 expression and immunomodulatory factors in various cancer types. We found that in most cancers, ACSM6 was positively correlated with a variety of immunomodulatory factors. However, in BLCA, ACSM6 was negatively correlated with a variety of immunomodulatory factors, including chemokines, receptors, MHC, immunosuppressants, and immunoactivators. Subsequently, we examined the correlation between ACSM6 expression and several pivotal immune checkpoints. Notably, negative associations were observed between ACSM6 and four immune checkpoints in BLCA: CTLA-4, PD-L1, PD-1, and LAG-3 (Figures 1B–E). Furthermore, we found that ACSM6 was inversely related to the ESTIMATE, immune, and matrix scores in BLCA (Figures 2A–C). In summary, ACSM6 is a potential biomarker for predicting tumor microenvironment status in BLCA. In BLCA, high ACSM6 expression may lead to a non-inflammatory TME because of decreased immunomodulatory factors, immune cells, immune checkpoints, and stromal cells in the TME.

### ACSM6 is related to the non-inflammatory tumor microenvironment of BLCA

**FIGURE 3:**
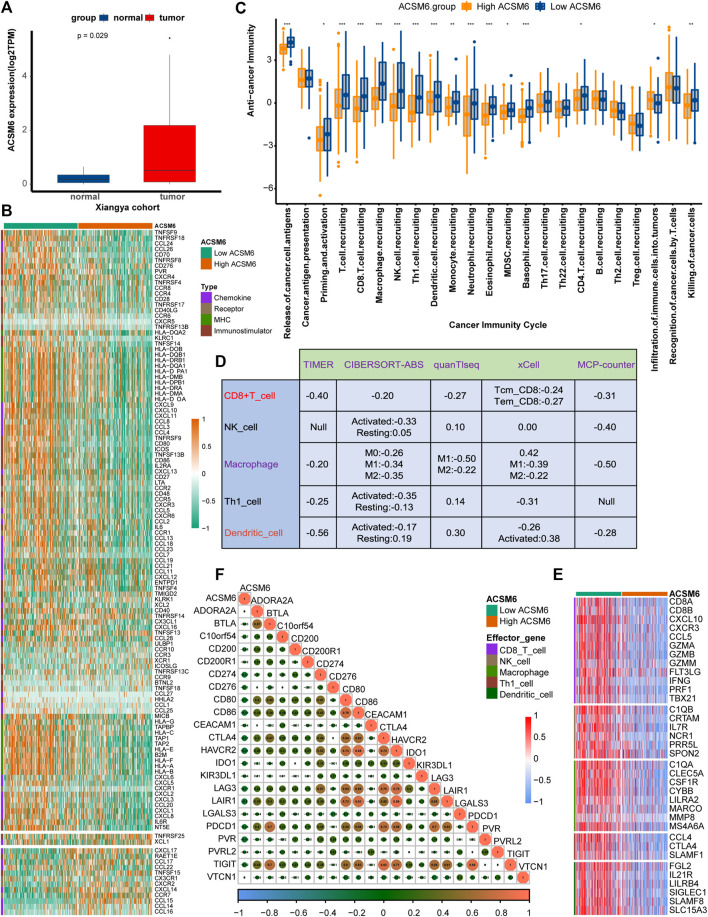
In BLCA, the tumor immune microenvironment correlates with ACSM6 expression. **(A)** ACSM6 was highly expressed in the Xiangya cohort. **(B)** ACSM6 expression is different in different tissues in BLCA. **(C)** ACSM6 expression is different in various steps of the anti-tumor immune cycle. **(D)** ACSM6 expression correlates with infiltration levels of five tumor-infiltrating immune cell types (TIICs), as measured by various algorithms. **(E)** High- and low-ACSM6 tissues in BLCA show differential expression of effector genes of the five TIICs mentioned above. **(F)** ACSM6 expression correlates with 20 inhibitory immune checkpoints in BLCA.

ACSM6 was highly expressed in the Xiangya cohort (Figure 3A) and was negatively correlated with multiple immunomodulatory factors (Figure 3B). Most chemokines, including CCL24, CCL26, CXCL9, CXCL10, and CCL8, were significantly reduced in the high ACSM6 group, and immune activators, including TNESF9, TNFRSF18, and TNFRSF8, were negatively correlated with ACSM6. Vast major steps in the tumor immune cycle in the high ACSM6 group were downregulated, including cancer cell antigen release, immune cell activation and recruitment, and cancer cell killing (Figure 3C).

**FIGURE 4:**
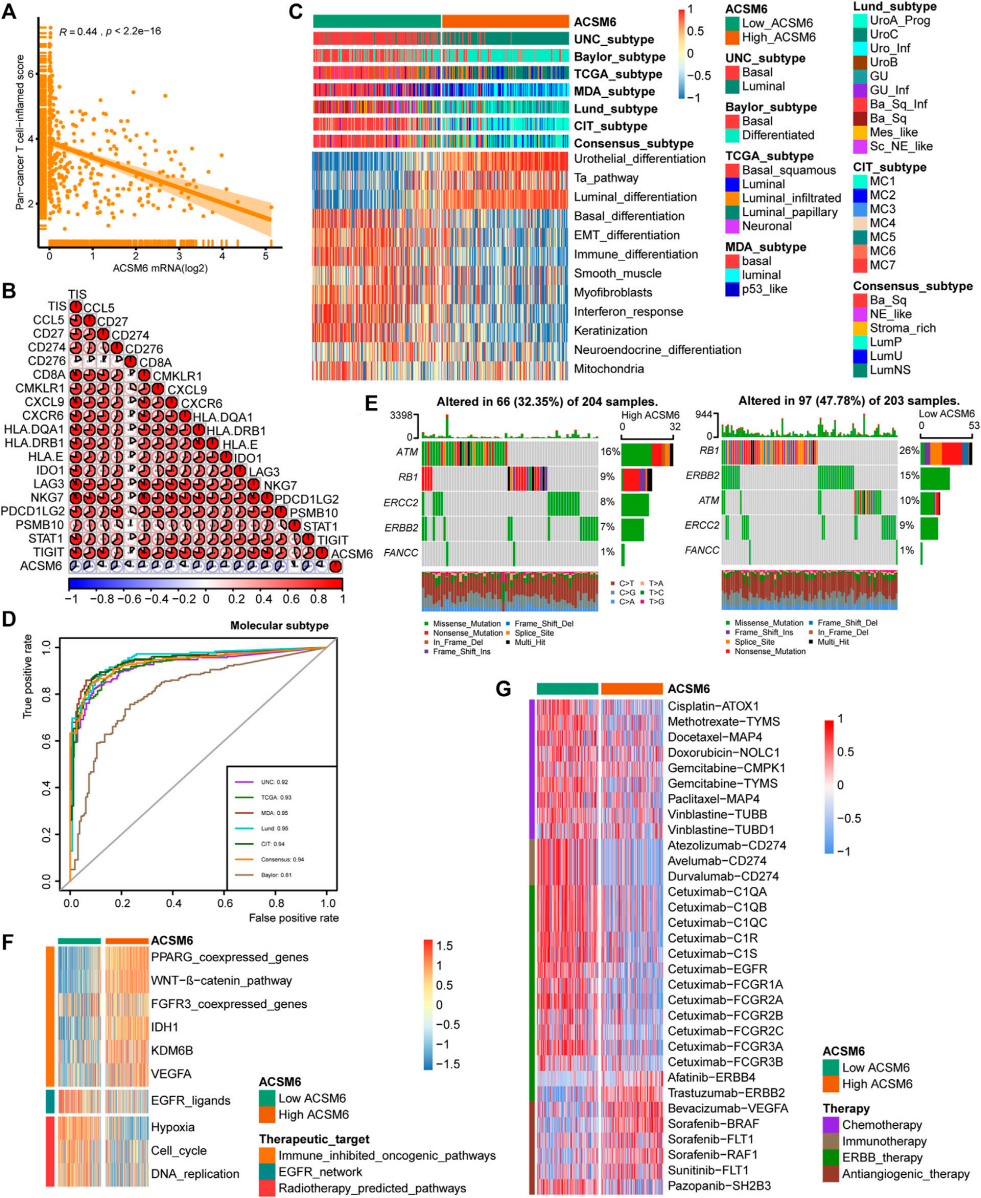
In BLCA, ACSM6 expression predicts molecular subtype and response to multiple therapies. **(A, B)** ACSM6 expression correlates with tumor immune subtype and their corresponding effector genes. **(C)** ACSM6 expression correlates with molecular subtypes as defined by seven different subtyping systems. **(D)** ROC analysis demonstrates the prediction accuracy of ACSM6 for molecular subtypes using different systems. **(E)** Mutational profiles of neoadjuvant chemotherapy-related genes differ between low- and high-ACSM6 tissues. **(F)** ACSM6 expression correlates with enrichment scores of therapeutic signatures. **(G)** ACSM6 expression correlates with drug-target genes for various therapies.

To further verify the relation between ACSM6 and TILs in the TME, we used five independent algorithms to calculate the infiltration levels of TILs. Results showed that ACSM6 expression negatively had to do with the infiltration levels of NK cells, CD8^+^ T cells, macrophages, Th1 cells, and dendritic cells (Figure 3D). Additionally, ACSM6 negatively correlated with TIL effector genes, including NK cells, CD8^+^ T cells, macrophages, dendritic cell-related genes, and Th1 cells (Figure 3E). Furthermore, the relationship between ACSM6 and immune checkpoint inhibitors in the TME was explored, which showed that ACSM6 negatively had to do with the most common immune checkpoint inhibitors, including C10orf54, CD86, CTLA4, HAVCR2, LAG-3, and PVR (Figure 3F). Additionally, ACSM6 negatively had to do with the TIS and its related TIS effector genes (Figures 4A, B). Collectively, these results demonstrate that ACSM6 promotes a non-inflammatory TME in BLCA.

### ACSM6 predicts molecular subtypes and drug sensitivity

Notably BLCA is a highly heterogeneous tumor and different molecular subtypes exhibit different sensitivities to different treatment regimens. Consequently, we distinguished the expression of ACSM6 among the BLCA molecular subtypes in TCGA. As shown in Figure 4C, patients with low ACSM6 expression developed the basal subtype, which characterized in EMT differentiation, basal differentiation, keratinization, and immune differentiation. Conversely, people with high ACSM6 expression were classified into the luminal/differentiated subtype, exhibiting Ta pathway and urothelial and supraluminal differentiation. Furthermore, ROC analysis was employed to evaluate the predictive precision of ACSM6 for molecular subtypes, with the area under the ROC curve ranging from 0.81 to 0.95 (Figure 4D).

Additional investigation of the association between ACSM6 and neoadjuvant chemotherapy (NAC) revealed that the low expression group of ACSM6 better turned to carry mutations related to NAC, such as ERBB2 (15%), RB1 (26%) and ATM (10%). Additionally, the high expression group of ACSM6 had higher mutation rates in ATM (16%), RB1 (9%), and ERBB2 (8%) (Figure 4E). Importantly, RB1 chemotherapy-related mutations were significantly higher in the low ACSM6 group, indicating that tumors with low ACSM6 expression were more likely to be sensitive to NAC. In addition, in the low ACSM6 group, the radiotherapy prediction pathway and EGFR ligand enrichment scores were higher (Figure 4F). Furthermore, the high ACSM6 group exhibited significantly higher enrichment scores for some immunosuppressive and carcinogenic pathways, including IDH1, WNT-β-catenin pathway, and PPARG co-expressed genes, suggesting the presence of a non-inflammatory TME in BLCA. Moreover, we utilized the Drug Bank database to identify the sensitivity of different groups to various therapies. Our results indicated that the low ACSM6 group was more responsive to immunotherapy and ERBB therapy, while the high ACSM6 group was more responsive to anti-angiogenesis therapy (Figure 4G). Overall, patients with low ACSM6 expression could be treated with adjuvant chemotherapy, neoadjuvant chemotherapy, immunotherapy, or ERBB.

### Validation of ACSM6 in Xiangya cohort

**FIGURE 5:**
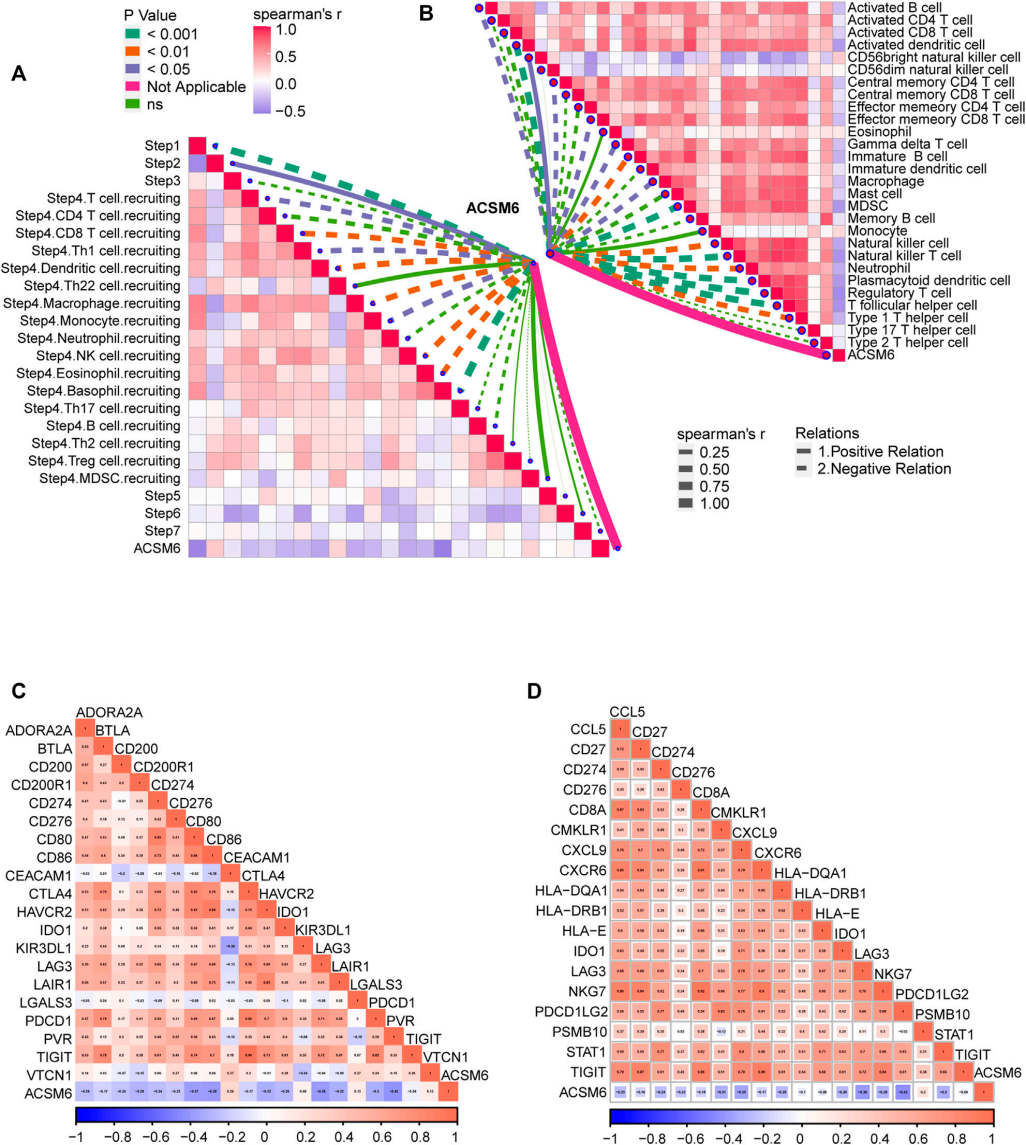
Validation of the prediction accuracy of ACSM6 for molecular subtypes and response to therapies in the Xiangya Cohort. **(A)** ACSM6 expression correlates with different steps of the anti-cancer immune cycle. **(B)** ACSM6 expression correlates with multiple ssGSEA immune cells. **(C)** ACSM6 expression correlates with 20 immune checkpoints. **(D)** ACSM6 expression correlates with TIS-related genes.

**FIGURE 6:**
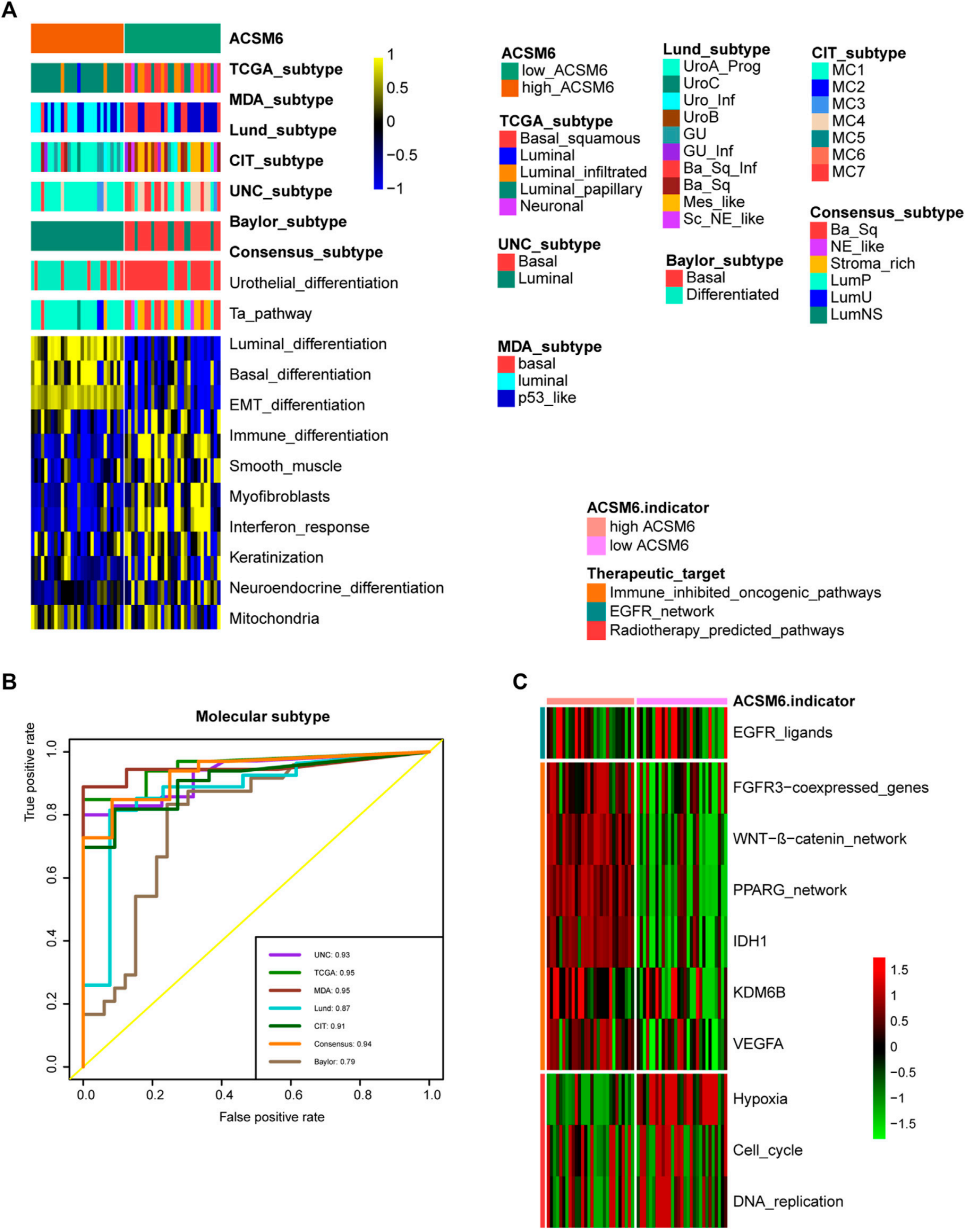
ACSM6 expression correlates with TIS-related effector genes. **(A)** ACSM6 expression correlates with seven different molecular subtyping systems. **(B)** The molecular subtype prediction accuracy of ACSM6 is evaluated. **(C)** ACSM6 expression correlates with enrichment scores of therapeutic signatures.

We performed additional analyses to explore the clinical significance of ACSM6 expression in the Xiangya cohort. ACSM6 was negatively associated with multiple key steps in the anticancer immune cycle, particularly the release of cancer cell antigens and the recruitment of immune cells (Figure 5A). Similarly, ACSM6 was negatively associated with various ssGSEA immune cells including activated dendritic cells, macrophages, natural killer cells, regulatory T cells, and T follicular helper cells (Figure 5B). Subsequently, ACSM6 levels were negatively associated with immune checkpoints in the Xiangya cohort (Figure 5C). Additionally, it was confirmed that ACSM6 was negatively related to the expression of effector genes in T cell activation scores, including CXCL9, LAG-3, and PDCD1LG2 (Figure 5D). In good line with the prediction of BLCA subtypes by TCGA, the high ACSM6 group in the Xiangya cohort better turned to be the luminal subtype, while the low ACSM6 group better turned to be the basal subtype (Figure 6A). Furthermore, ACSM6 exhibited higher accuracy in predicting the BLCA molecular subtypes (Figure 6B). Notably, the high ACSM6 group better turned to be effected by immunosuppressive tumor therapy (Figure 6C).

### ACSM6 predicts the clinical efficacy of ICB

**FIGURE 7:**
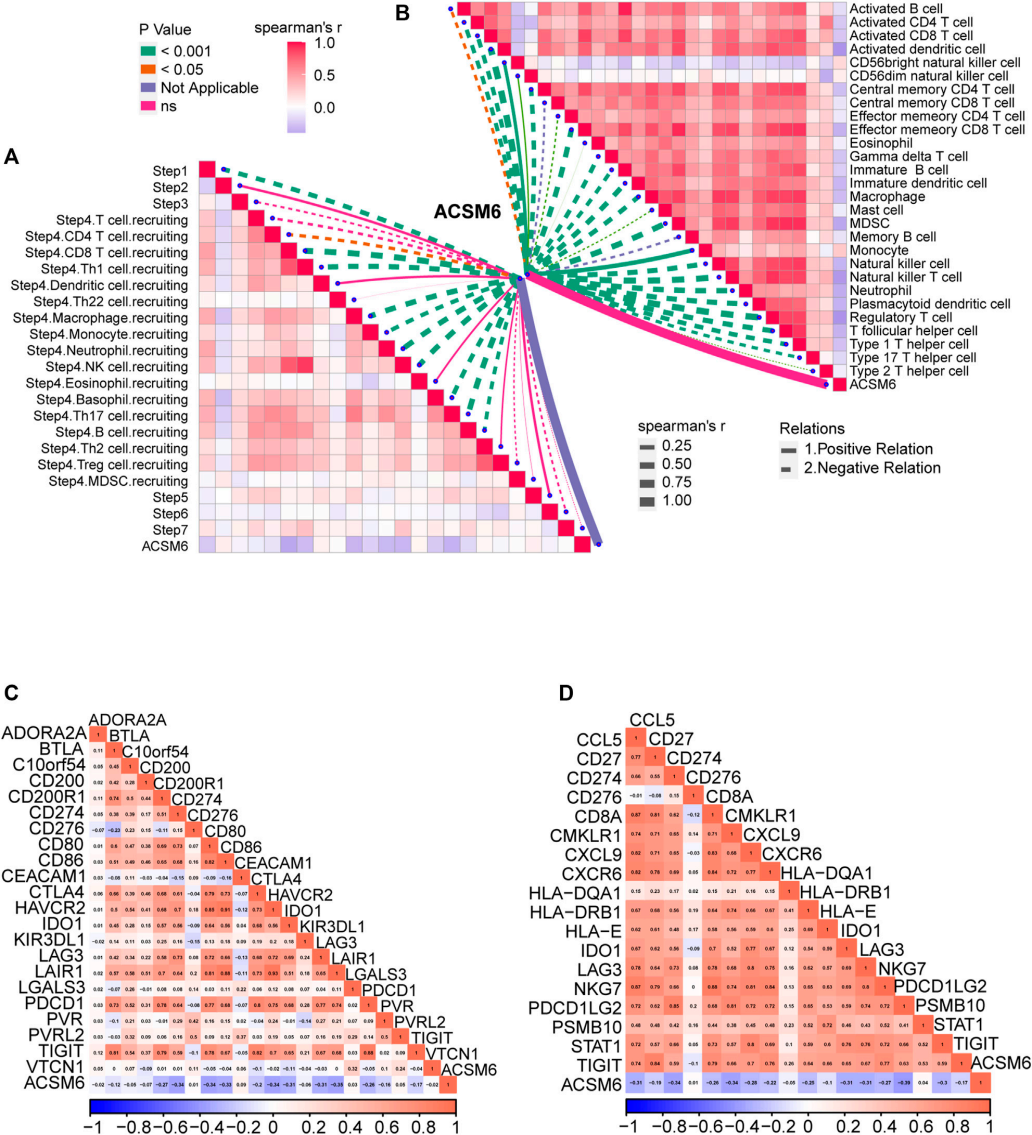
Validation of prediction for low immune infiltration and molecular subtypes by ACSM6 in the IMvigor210 cohort. **(A)** ACSM6 expression correlates with different steps of the anti-cancer immune cycle. **(B)** ACSM6 expression correlates with several immune-related cells. **(C)** ACSM6 expression correlates with immune checkpoint inhibitors effector genes. **(D)** ACSM6 expression correlates with TIS-related genes.

**FIGURE 8:**
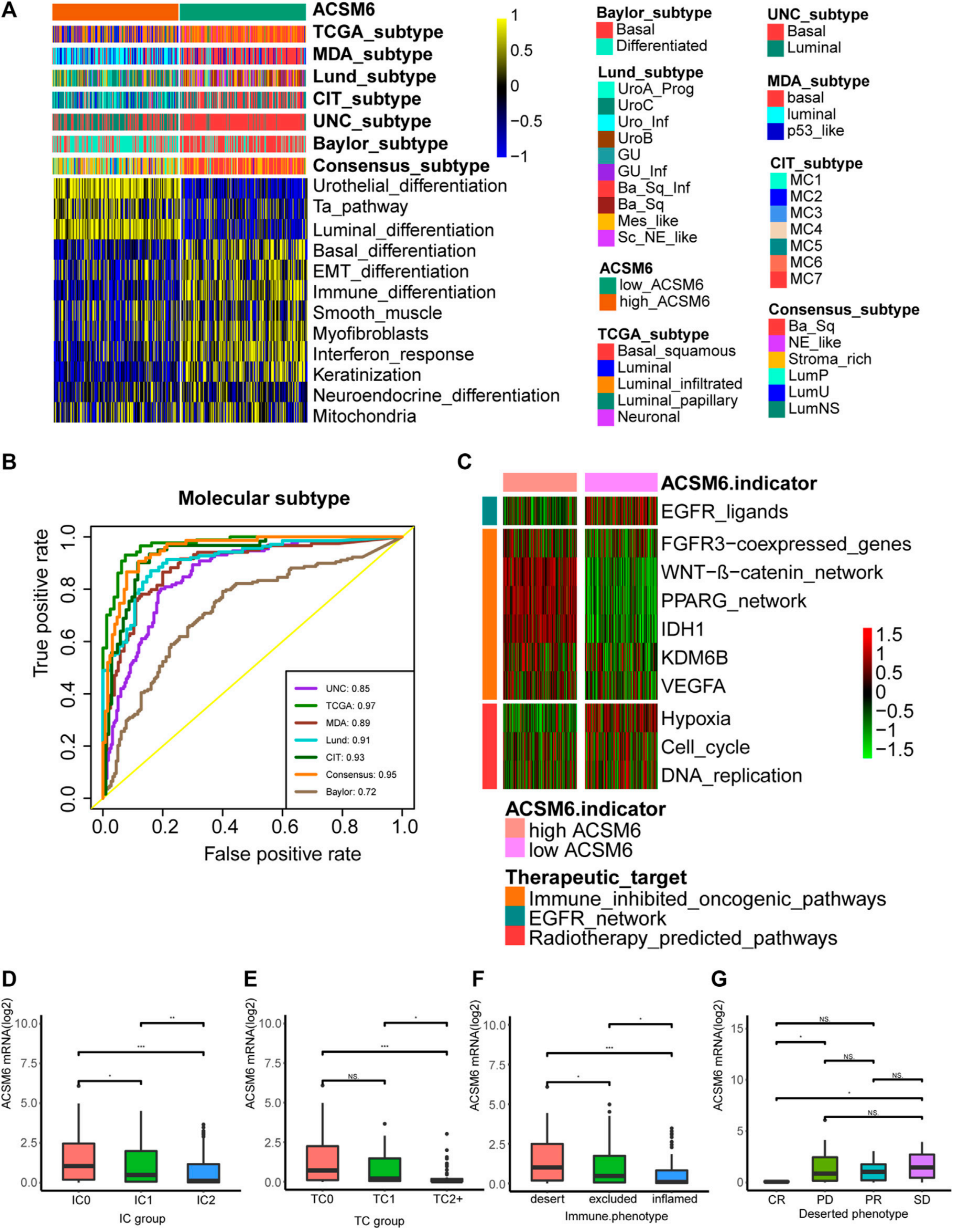
Correlations between ACSM6 and seven molecular subtype systems, three immune phenotypes and the clinical response of tumor immunotherapy in the desert group. **(A, B)** ACSM6 expression correlates with seven different molecular subtyping systems, and its prediction accuracy is evaluated by ROC analysis. **(C)** ACSM6 expression correlates with enrichment scores of therapeutic signatures. **(D)** ACSM6 expression shows differential expression in different immune checkpoint groups. **(E)** ACSM6 expression shows differential expression in different IC groups. **(F)** ACSM6 expression shows differential expression in three immune phenotypes: the desert, excluded, and inflamed. **(G)** ACSM6 expression correlates with the clinical response of tumor immunotherapy in the desert group.

We investigated the predictive value of ACSM6 for the clinical response to ICB in the IMvigor210 cohort. In the Xiangya cohort, we observed a negative correlation between ACSM6 expression and several critical steps in the cancer immune cycle, which was consistent with our findings (Figure 7A), suggesting that when ACSM6 is highly expressed, TIL infiltration in TME is downregulated (Figure 7B). Additionally, a negative correlation was observed between ACSM6 expression and the expression of several ICI and TIS genes (Figures 7C, D). Furthermore, high ACSM6 expression predicted the luminal subtype of BLCA, which was in line with the findings in TCGA cohort (Figure 8A). The area under the ROC curve for predicting molecular subtypes was between 0.72 and 0.97 (Figure 8B). In addition, we found that the enrichment score for the radiotherapy prediction pathway in the low ACSM6 group was higher (Figure 8C).

We conducted immunohistochemistry (IHC) on the IMvigor210 cohort to identify PD-L1 expression in immune and cancer cells. We classified the immune and cancer cells into three groups based on their PD-L1 expression levels. We observed significant ACSM6 expression in the TC0 and IC0 groups (Figures 8D, E). In addition, ACSM6 was highly expressed in the non-inflammatory TME compared to that in the inflammatory TME (Figure 8F). Furthermore, we compared ACSM6 expression in different ICB clinical responses. In spite of lower ACSM6 expression in patients with complete remission (CR) in the non-inflammatory TME, ACSM6 expression was higher in patients with progressive disease (PD), stable disease (SD), and partial remission (PR). However, no significant differences were observed between these groups (Figure 8G).

## Discussion

This study found that ACSM6 may act as a potential molecular biomarker for assessing the tumor microenvironment status in various types of cancer, particularly in BLCA and thymoma, and may lead to the formation of a non-inflammatory TME in BLCA. Moreover, ACSM6 can be used to predict the BLCA molecular subtypes. Low ACSM6 expression was observed in the basal subtype and exhibited higher sensitivity to ICB immune infiltration levels. Furthermore, patients with low ACSM6 expression were likely to respond better to neoadjuvant chemotherapy, adjuvant chemotherapy, and ERBB treatment.

ACSM6 is a member 6 of the acyl-CoA synthase middle-chain family; as a new member, no related research has been conducted. However, ACSM3, which belongs to the same family, has been found to be associated with tumor progression. The expression of ACSM3 is significantly reduced in hepatocellular carcinoma tissues and is associated with the late stage and poor survival rates of hepatocellular carcinoma (Boomgaarden et al., 2009). Overexpression of ACSM3 is able to weaken the migration and invasion of hepatocellular carcinoma cells (Ruan et al., 2017; Ruan et al., 2021). ACSM3 also has been reported to suppresses the pathogenesis of high-grade serous ovarian carcinoma via promoting AMPK activity (Yang et al., 2022). In addition, krüppel-like factor 10 can upregulate ACSM3 via the PI3K/Akt signaling pathway to inhibit the malignant progression of melanoma (Zhao et al., 2022). These reports indicate that ACSM3 has a high probability of inhibiting tumors. ACSM1 (Guo et al., 2022) and ACSM5 (Ruan et al., 2021) have been reported to be associated with the progression of prostate and thyroid cancers, respectively. ACSM1 is usually used as a molecular marker of apocrine carcinoma of the breast (Celis et al., 2009). ACSM4 is associated with poor prognosis of triple-negative breast cancer (Alsaleem et al., 2020). However, there are few related studies.

This study has some limitations. First, all results were from bioinformatics analyses, and no *in vivo* or *in vitro* experiments were conducted to investigate the possible mechanisms. Second, although our results were robust in the Xiangya cohort, the small sample size (57 patients) cannot be ignored. Third, grouping into high- and low-expression categories was based on the median ACSM6 mRNA expression, which might have certain limitations, and an ideal cutoff value was not identified. Therefore, this must be verified using additional tumor tissue data and experiments.

## Conclusion

Our study showed that the presence of ACSM6 plays the promotion to the development of a non-inflammatory TME in BLCA, which in turn results in resistance to tumor immunotherapy. ACSM6 may be a predictor of BLCA molecular subtypes, suggesting a better prognosis treatment.

## Data Availability

The original contributions presented in the study are included in the article/Supplementary Material, further inquiries can be directed to the corresponding authors.

## References

[B1] Alsaleem M. A. Ball G. Toss M. S. Raafat S. Aleskandarany M. Joseph C. (2020). A novel prognostic two-gene signature for triple negative breast cancer. Mod. Pathol. 33 (11), 2208–2220. 10.1038/s41379-020-0563-7 32404959

[B2] Auslander N. Zhang G. Lee J. S. Frederick D. T. Miao B. Moll T. (2018). Robust prediction of response to immune checkpoint blockade therapy in metastatic melanoma. Nat. Med. 24 (10), 1545–1549. 10.1038/s41591-018-0157-9 30127394 PMC6693632

[B3] Ayers M. Lunceford J. Nebozhyn M. Murphy E. Loboda A. Kaufman D. R. (2017). IFN-γ-related mRNA profile predicts clinical response to PD-1 blockade. J. Clin. Invest. 127 (8), 2930–2940. 10.1172/JCI91190 28650338 PMC5531419

[B4] Becht E. Giraldo N. A. Lacroix L. Buttard B. Elarouci N. Petitprez F. (2016). Erratum to: Estimating the population abundance of tissue-infiltrating immune and stromal cell populations using gene expression. Genome Biol. 17 (1), 249. 10.1186/s13059-016-1113-y 27908289 PMC5134277

[B5] Boomgaarden I. Vock C. Klapper M. Döring F. (2009). Comparative analyses of disease risk genes belonging to the acyl-CoA synthetase medium-chain (ACSM) family in human liver and cell lines. Biochem. Genet. 47 (9-10), 739–748. 10.1007/s10528-009-9273-z 19634011

[B6] Cai Z. Chen J. Yu Z. Li H. Liu Z. Deng D. (2023). BCAT2 shapes a noninflamed tumor microenvironment and induces resistance to anti-PD-1/PD-L1 immunotherapy by negatively regulating proinflammatory chemokines and anticancer immunity. Adv. Sci. (Weinh) 10 (8), e2207155. 10.1002/advs.202207155 36642843 PMC10015882

[B7] Catto J. W. F. Downing A. Mason S. Wright P. Absolom K. Bottomley S. (2021). Quality of life after bladder cancer: A cross-sectional survey of patient-reported outcomes. Eur. Urol. 79 (5), 621–632. 10.1016/j.eururo.2021.01.032 33581875 PMC8082273

[B8] Celis J. E. Cabezón T. Moreira J. M. A. Gromov P. Gromova I. Timmermans-Wielenga V. (2009). Molecular characterization of apocrine carcinoma of the breast: Validation of an apocrine protein signature in a well-defined cohort. Mol. Oncol. 3 (3), 220–237. 10.1016/j.molonc.2009.01.005 19393583 PMC5527852

[B9] Chang E. Weinstock C. Zhang L. Charlab R. Dorff S. E. Gong Y. (2021). FDA approval summary: Enfortumab vedotin for locally advanced or metastatic urothelial carcinoma. Clin. Cancer Res. 27 (4), 922–927. 10.1158/1078-0432.CCR-20-2275 32962979

[B10] Charoentong P. Finotello F. Angelova M. Mayer C. Efremova M. Rieder D. (2017). Pan-cancer immunogenomic analyses reveal genotype-immunophenotype relationships and predictors of response to checkpoint blockade. Cell. Rep. 18 (1), 248–262. 10.1016/j.celrep.2016.12.019 28052254

[B11] Chen D. S. Mellman I. (2013). Oncology meets immunology: The cancer-immunity cycle. Immunity 39 (1), 1–10. 10.1016/j.immuni.2013.07.012 23890059

[B12] Feng D. Liu S. Li D. Han P. Wei W. (2020). Analysis of conventional versus advanced pelvic floor muscle training in the management of urinary incontinence after radical prostatectomy: A systematic review and meta-analysis of randomized controlled trials. Transl. Androl. Urol. 9 (5), 2031–2045. 10.21037/tau-20-615 33209667 PMC7658159

[B13] Feng D. Shi X. Zhang F. Xiong Q. Wei Q. Yang L. (2022a). Mitochondria dysfunction-mediated molecular subtypes and gene prognostic index for prostate cancer patients undergoing radical prostatectomy or radiotherapy. Front. Oncol. 12, 858479. 10.3389/fonc.2022.858479 35463369 PMC9019359

[B14] Feng D. Tang C. Liu S. Yang Y. Han P. Wei W. (2022b). Current management strategy of treating patients with erectile dysfunction after radical prostatectomy: A systematic review and meta-analysis. Int. J. Impot. Res. 34 (1), 18–36. 10.1038/s41443-020-00364-w 33099581

[B15] Finotello F. Mayer C. Plattner C. Laschober G. Rieder D. Hackl H. (2019). Molecular and pharmacological modulators of the tumor immune contexture revealed by deconvolution of RNA-seq data. Genome Med. 11 (1), 34. 10.1186/s13073-019-0638-6 31126321 PMC6534875

[B16] Grivas P. Agarwal N. Pal S. Kalebasty A. R. Sridhar S. S. Smith J. (2021). Avelumab first-line maintenance in locally advanced or metastatic urothelial carcinoma: Applying clinical trial findings to clinical practice. Cancer Treat. Rev. 97, 102187. 10.1016/j.ctrv.2021.102187 33839438

[B17] Guo Y. Ren C. Huang W. Yang W. Bao Y. (2022). Oncogenic ACSM1 in prostate cancer is through metabolic and extracellular matrix-receptor interaction signaling pathways. Am. J. Cancer Res. 12 (4), 1824–1842. 35530294 PMC9077067

[B18] Hu J. Chen J. Ou Z. Chen H. Liu Z. Chen M. (2022). Neoadjuvant immunotherapy, chemotherapy, and combination therapy in muscle-invasive bladder cancer: A multi-center real-world retrospective study. Cell. Rep. Med. 3 (11), 100785. 10.1016/j.xcrm.2022.100785 36265483 PMC9729796

[B19] Hu J. Othmane B. Yu A. Li H. Cai Z. Chen X. (2021a). 5mC regulator-mediated molecular subtypes depict the hallmarks of the tumor microenvironment and guide precision medicine in bladder cancer. BMC Med. 19 (1), 289. 10.1186/s12916-021-02163-6 34836536 PMC8627095

[B20] Hu J. Yu A. Othmane B. Qiu D. Li H. Li C. (2021b). Siglec15 shapes a non-inflamed tumor microenvironment and predicts the molecular subtype in bladder cancer. Theranostics 11 (7), 3089–3108. 10.7150/thno.53649 33537076 PMC7847675

[B21] Li B. Severson E. Pignon J. C. Zhao H. Li T. Novak J. (2016). Comprehensive analyses of tumor immunity: Implications for cancer immunotherapy. Genome Biol. 17 (1), 174. 10.1186/s13059-016-1028-7 27549193 PMC4993001

[B22] Li T. Fu J. Zeng Z. Cohen D. Li J. Chen Q. (2020). TIMER2.0 for analysis of tumor-infiltrating immune cells. Nucleic Acids Res. 48 (W1), W509–W514. 10.1093/nar/gkaa407 32442275 PMC7319575

[B23] Liu S. Chen X. Lin T. (2022). Emerging strategies for the improvement of chemotherapy in bladder cancer: Current knowledge and future perspectives. J. Adv. Res. 39, 187–202. 10.1016/j.jare.2021.11.010 35777908 PMC9263750

[B24] Liu Z. Tang Q. Qi T. Othmane B. Yang Z. Chen J. (2021). A robust hypoxia risk score predicts the clinical outcomes and tumor microenvironment immune characters in bladder cancer. Front. Immunol. 12, 725223. 10.3389/fimmu.2021.725223 34484235 PMC8415032

[B25] Mariathasan S. Turley S. J. Nickles D. Castiglioni A. Yuen K. Wang Y. (2018). TGFβ attenuates tumour response to PD-L1 blockade by contributing to exclusion of T cells. Nature 554 (7693), 544–548. 10.1038/nature25501 29443960 PMC6028240

[B26] Morales-Barrera R. Suárez C. González M. Valverde C. Serra E. Mateo J. (2020). The future of bladder cancer therapy: Optimizing the inhibition of the fibroblast growth factor receptor. Cancer Treat. Rev. 86, 102000. 10.1016/j.ctrv.2020.102000 32203842

[B27] Newman A. M. Liu C. L. Green M. R. Gentles A. J. Feng W. Xu Y. (2015). Robust enumeration of cell subsets from tissue expression profiles. Nat. Methods 12 (5), 453–457. 10.1038/nmeth.3337 25822800 PMC4739640

[B28] Ruan H. Y. Yang C. Tao X. M. He J. Wang T. Wang H. (2017). Downregulation of ACSM3 promotes metastasis and predicts poor prognosis in hepatocellular carcinoma. Am. J. Cancer Res. 7 (3), 543–553. 28401010 PMC5385642

[B29] Ruan X. Tian M. Kang N. Ma W. Zeng Y. Zhuang G. (2021). Genome-wide identification of m6A-associated functional SNPs as potential functional variants for thyroid cancer. Am. J. Cancer Res. 11 (11), 5402–5414. 34873468 PMC8640822

[B30] Sassoli C. Pierucci F. Zecchi-Orlandini S. Meacci E. (2019). Sphingosine 1-phosphate (S1P)/S1P receptor signaling and mechanotransduction: Implications for intrinsic tissue repair/regeneration. Int. J. Mol. Sci. 20 (22), 5545. 10.3390/ijms20225545 31703256 PMC6888058

[B31] Sung H. Ferlay J. Siegel R. L. Laversanne M. Soerjomataram I. Jemal A. (2021). Global cancer statistics 2020: GLOBOCAN estimates of incidence and mortality worldwide for 36 cancers in 185 countries. CA Cancer J. Clin. 71 (3), 209–249. 10.3322/caac.21660 33538338

[B32] Svatek R. S. Hollenbeck B. K. Holmäng S. Lee R. Kim S. P. Stenzl A. (2014). The economics of bladder cancer: Costs and considerations of caring for this disease. Eur. Urol. 66 (2), 253–262. 10.1016/j.eururo.2014.01.006 24472711

[B33] van Rhijn B. W. G. Burger M. Lotan Y. Solsona E. Stief C. G. Sylvester R. J. (2009). Recurrence and progression of disease in non-muscle-invasive bladder cancer: From epidemiology to treatment strategy. Eur. Urol. 56 (3), 430–442. 10.1016/j.eururo.2009.06.028 19576682

[B34] Xu L. Deng C. Pang B. Zhang X. Liu W. Liao G. (2018). Tip: A web server for resolving tumor immunophenotype profiling. Cancer Res. 78 (23), 6575–6580. 10.1158/0008-5472.CAN-18-0689 30154154

[B35] Yang X. Wu G. Zhang Q. Chen X. Li J. Han Q. (2022). ACSM3 suppresses the pathogenesis of high-grade serous ovarian carcinoma via promoting AMPK activity. Cell. Oncol. (Dordr). 45 (1), 151–161. 10.1007/s13402-021-00658-1 35124784 PMC12978076

[B36] Zhao Z. Zhan Y. Jing L. Zhai H. (2022). KLF10 upregulates ACSM3 via the PI3K/Akt signaling pathway to inhibit the malignant progression of melanoma. Oncol. Lett. 23 (6), 175. 10.3892/ol.2022.13295 35497935 PMC9019859

